# Combining QTL-seq and linkage mapping to fine map a candidate gene in *qCTS6* for cold tolerance at the seedling stage in rice

**DOI:** 10.1186/s12870-021-03076-5

**Published:** 2021-06-19

**Authors:** Luomiao Yang, Jingguo Wang, Zhenghong Han, Lei Lei, Hua Long Liu, Hongliang Zheng, Wei Xin, Detang Zou

**Affiliations:** Key Laboratory of Germplasm Enhancement, Physiology and Ecology of Food Crops in Cold Region, Ministry of Education, Northeast Agricultural University, Harbin, 150030 China

**Keywords:** *Oryza sativa* L., Cold tolerance, QTL-Seq, Linkage-mapping, Seedling stage

## Abstract

**Background:**

Cold stress caused by low temperatures is an important factor restricting rice production. Identification of cold-tolerance genes that can stably express in cold environments is crucial for molecular rice breeding.

**Results:**

In this study, we employed high-throughput quantitative trait locus sequencing (QTL-seq) analyses in a 460-individual F_2:3_ mapping population to identify major QTL genomic regions governing cold tolerance at the seedling stage in rice. A novel major QTL (*qCTS6*) controlling the survival rate (SR) under low-temperature conditions of 9°C/10 days was mapped on the 2.60-Mb interval on chromosome 6. Twenty-seven single-nucleotide polymorphism (SNP) markers were designed for the *qCST6* region based on re-sequencing data, and local QTL mapping was conducted using traditional linkage analysis. Eventually, we mapped *qCTS6* to a 96.6-kb region containing 13 annotated genes, of which seven predicted genes contained 13 non-synonymous SNP loci. Quantitative reverse transcription PCR analysis revealed that only Os06g0719500, an *OsbZIP54* transcription factor, was strongly induced by cold stress. Haplotype analysis confirmed that +376 bp (T>A) in the *OsbZIP54* coding region played a key role in regulating cold tolerance in rice.

**Conclusion:**

We identified *OsbZIP54* as a novel regulatory gene associated with rice cold-responsive traits, with its Dongfu-104 allele showing specific cold-induction expression serving as an important molecular variation for rice improvement. This result is expected to further exploration of the genetic mechanism of rice cold tolerance at the seedling stage and improve cold tolerance in rice varieties by marker-assisted selection.

**Supplementary Information:**

The online version contains supplementary material available at 10.1186/s12870-021-03076-5.

## Background

Rice (*Oryza sativa* L.) is an important food crop that has adapted to a tropical climate. Therefore, cold damage of rice at high latitudes and high altitudes has become an important factor restricting its production [1]. Specifically during the early stage of vegetative growth, chilling directly causes physiological damage to seedlings, thereby hindering the vegetative formation of the plants and ultimately affecting yield [2]. Therefore, the key to solving this problem is to explore cold tolerance (CT) genes with strong advantages that contribute alleles to ultimately improve plant CT.

CT in rice is a quantitative trait controlled by multiple genetic and environmental factors. In the previous 20 years, numerous double haploid lines, back-cross inbred lines, recombinant inbred lines, and near-isogenic lines have been developed using CT varieties, such as Dongxiang wild rice [3], KunmingXiaobaigu [4], LijiangXintuanheigu [5], Koshihikari [6], M202 [7], and Norin-PL8 [8], as donors, and >250 CT-related quantitative trait loci (QTLs) have been identified on 12 chromosomes [9]. To date, >80 QTLs for CT at the seedling stage (CTS) in rice have been identified [2, 10–12]. Moreover, most QTLs that are linked with CTS are located within a range of 10 cM to 30 cM through primary populations, making it difficult to perform molecular-marker-assisted breeding. Thus, the key to solving this problem is to construct a fine-mapping population. To date, *qCTS4* [13], *qCtss11* [14], *qSCT1* [15], *qSCT11* [15], and *qLOP2/qPSR2-1* [16] have been fine-mapped, with only seven genes responding to CTS isolated by map-based cloning (*COLD1*, *qCTS-9*, *qPSR10*, *bZIP73*, *HAN1*, *OsLTPL159* and *qCST10*). *COLD1* [17] encodes a G protein signal regulator, and its overexpression can significantly improve rice CT, whereas rice lines with no or low expression are cold-sensitive. The promoter region of *qCTS-9* [18] has an indel marker significantly associated with low-temperature tolerance, and its overexpression increases rice tolerance to low temperatures. *qPSR10* [19] was detected in a genome-wide association study, and its CT haplotype was found in japonica rice resources. *bZIP73* [1] has a single-nucleotide polymorphism (SNP) on the genome of japonica and indica rice, with this SNP determining the tolerance of rice to low temperatures. *HAN1* [20] regulates low-temperature response and CTS mediated by jasmonic acid and is a negative regulator of CTS. *OsLTPL159* [21] encodes a nonspecific lipid-transfer protein, the coding sequence (CDS) for which is located in the mapped region of *qCST10* [22]. These examples demonstrate that molecular regulation of CTS is extremely complex, and although these studies have laid the foundation for understanding the molecular mechanism of CT in early rice growth, dissecting the genetics behind CT in rice is still at its infancy. Identification of these QTLs by biparental cross-linkage mapping was a labor- and time-intensive process that included mapping the genotypes a large number of individuals in a segregated population; therefore, to obtain reliable QTLs, CTS improvement needs to be accomplished through fine mapping, gene cloning, functional verification, and finally application for molecular-marker-assisted breeding.

The rapid development of next-generation sequencing (NGS) technology has enabled the combination of bulked segregant analysis (BSA) with NGS to become an important method for mining QTLs and genes [23, 24]. Compared with traditional linkage mapping, QTL sequencing (QTL-seq) can improve work efficiency and provide high-density variation. QTL-seq has been successfully applied for many plants, including cucumber [25], soybean [26], rice [27], and tomato [28]. Although the technique enables rapid location of the major QTL, confidence interval (CI) resolution remains less than optimal. Therefore, researchers have had to combine QTL-seq with fine mapping [29] and RNA-seq [30] in order to identify candidate genes with a QTL. For example, *CaqSW1.1* [31] was mapped from 1.37 Mb to 35 kb by QTL-seq and classical QTL mapping to allow identification of final candidate genes. Additionally, four candidate genes (*SlCathB2, SlGST, SlUBC5,* and *SlARG1*) associated with heat tolerance were detected by QTL-seq and RNA-seq in tomato [28]. Thus, utilization of a combined method for identifying a major QTL interval via QTL-seq and fine mapping to mine target genes provides a new perspective.

In this study, we employed two strategies (QTL-seq and fine mapping) to identify the genes for CTS in rice. An F_2:3_ population derived from a cross between Dongnong430 (a cold-sensitive variety) and Dongfu104 (a strong CT variety) were used for QTL-seq analysis, and *qCTS6* was mapped in the 2.60-Mb genomic region on chromosome 6. Furthermore, we used a fine-mapping strategy to anchor *qCTS6* in the 96.6-kb interval, where 13 non-synonymous SNPs (nSNPs) were identified within the seven-gene intervals, which were predicted to result in an amino acid change. Quantitative reverse transcription (qRT)-PCR and haplotype analysis revealed that *OsbZIP54* was strongly induced by cold stress at the seedling stage, and that +376-bp (T>A)^Dongfu104^ can be considered a rare functional variation of japonica rice in northern China. These results revealed the mechanism by which cold sensitivity changes into CT in rice at the molecular level and improved the understanding of the mechanisms underlying CTS in rice.

## Results

### Screening and evaluation of the CT score

**Fig. 1:**
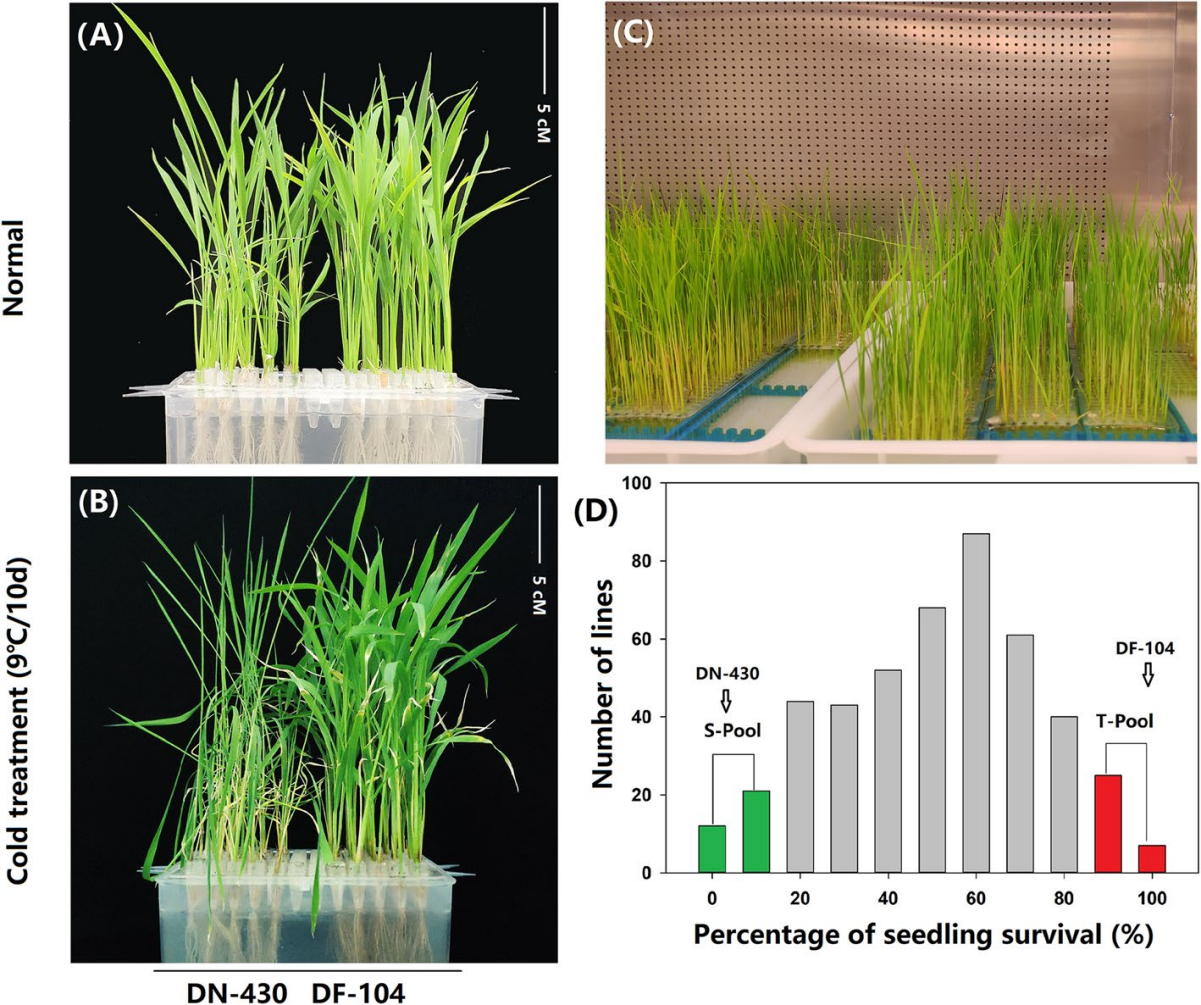
Phenotype analysis of CTS in rice. **A** Performance of DN430 and DF104 under normal conditions. **B** Performance of DN420 and DF104 under cold treatment (9°C/10 days). **C** Identification of CT in the F2 population in an artificial climate chamber. **D** Frequency distribution of SR variation measured among 460 mapping individuals of F2:3 populations

Two parental varieties, DN430 and DF104 (Fig. 1A, B) along with their 460 F_2:3_ lines (Fig. 1C), were evaluated under normal and cold conditions of 9°C/10 days. The SR of the CT-variety DF104 was significantly higher than that of cold-sensitive DN430 (Fig. 1B), indicating that DF104 had a stronger CT than DN430. In the F_2:3_ population, the absolute values of skewness and kurtosis associated with the SR were close to 1 (Table S1), indicating the data as suitable for QTL analysis. Moreover, the F_2:3_ population showed different SRs from 0 to 100, with Fig. 1D showing that among the 460 lines, 30 CT and 30 cold-sensitive lines were identified. These were then selected to prepare the CT (T-pool) and sensitive pools (S-pool), respectively, which were then used for DNA re-sequencing.

### Whole-genome re-sequencing and QTL-seq analysis

The average genome-coverage depth of the parents and two pools was 50×. When compared with the ‘Nipponbare’ reference genome, we obtained a total of 786,407 SNPs and 133,992 indels for four samples. After trimming and filtering, 521,630 SNPs and 85,824 indels were obtained (Table S2). From these SNPs, a total of 356,831 high-quality SNPs homozygous for each parent and showing polymorphism between the parents were developed for further QTL-seq analysis.

**Fig. 2:**
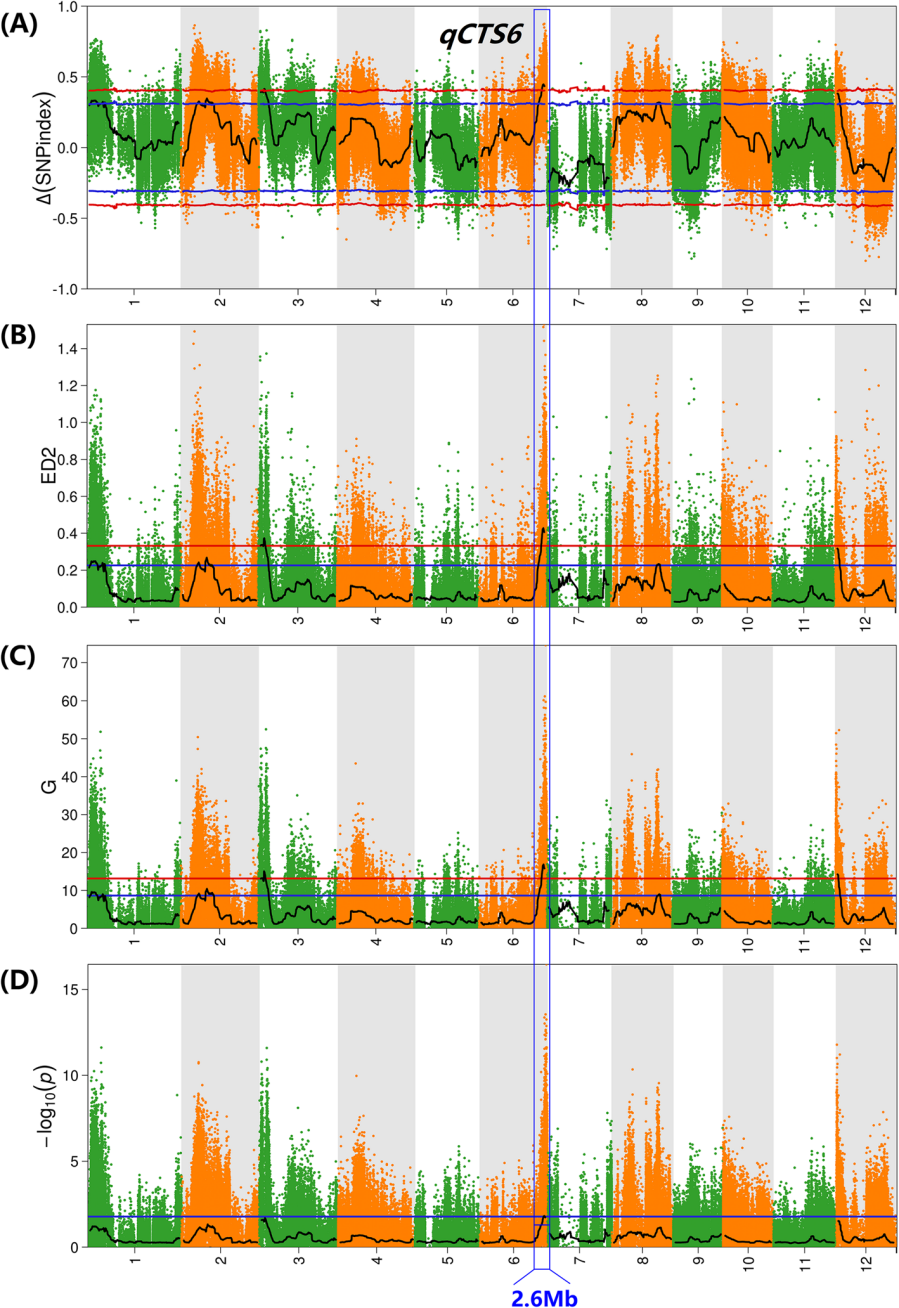
QTL analysis of CTS using QTL-seq. **A** Manhattan plot showing the distribution of Δ(SNP-index) on chromosomes, **B** the distribution of the square of the ED on the chromosomes, **C** the distribution of G-value on the chromosomes, and **D** the distribution of −log_10_(*P*-value) on the chromosomes based on Fisher’s exact test. The blue and red lines represent 95 and 99% CIs, respectively. The black lines are average values of the four algorithms and were drawn by sliding window analysis. The number on the horizontal coordinate represents the chromosome number

For all of the obtained SNPs, the ∆(SNP-Index) (Fig. 2A), Euclidean distance (ED) (Fig. 2B), G-value (Fig. 2C), and Fisher’s exact test (Fig. 2D) were used to intercept the association interval. We then identified the candidate regions associated with CTS in the rice genome (Table 1). For the ∆(SNP-Index), two major peaks on chromosomes 3 and 6 were identified for CTS (*P* < 0.01) spanning 2.58-Mb (1.50–4.08 Mb) and 3.24-Mb (28.00–31.24 Mb) intervals. The significant peak given by the ED algorithm completely covered the results returned by the ∆(SNP-Index) algorithm, and the two major intervals were subsequently named *qCTS3* and *qCTS6*. The G algorithm similarly captured the results of the first two algorithms and identified another new interval (0.00–2.42 Mb) in chromosome 12 (named *qCST12*). Only *qCTS6* was obtained when the four methods were applied. By comparing the association results of the four methods, their intersections of the genome regions were consistent with the association regions identified by Fisher’s exact test. A total of 2.60 Mb was found in the *qCTS6* interval. Notably, in the three algorithms, the peak value obtained by *qCTS6* was higher than that of *qCTS3* (Table S3), indicating a significant difference in the allele ratio between the two mixed pools. Therefore, *qCTS6* was considered the most significant CTS target for exploring candidate genes. According to statistical analysis, the *qCTS6* coding region harbored 407 SNPs and 18 indels (Table S4).

**Table 1:** QTLs conferring cold tolerance in four method identified using QTL-seq

Method	QTL name	Chr.	Start (bp)	End (bp)	Peak
Δ(SNP-index)	*qCTS3*	3	1500001	4080000	0.4153
	*qCTS6*	6	28000001	31248787	0.4468
ED-Value	*qCTS3*	3	1	4200000	0.3730
	*qCTS6*	6	27840001	31248787	0.4277
G-Value	*qCTS3*	3	80001	4180000	15.0450
	*qCTS6*	6	27860001	31248787	16.8494
	*qCTS12*	12	1	2420000	14.2366
Fish-*P*-Value	*qCTS6*	6	28440001	31040000	0.0359

*Chr.* chromosome

### Narrowing of *qCTS6* to a fine region

**Fig. 3:**
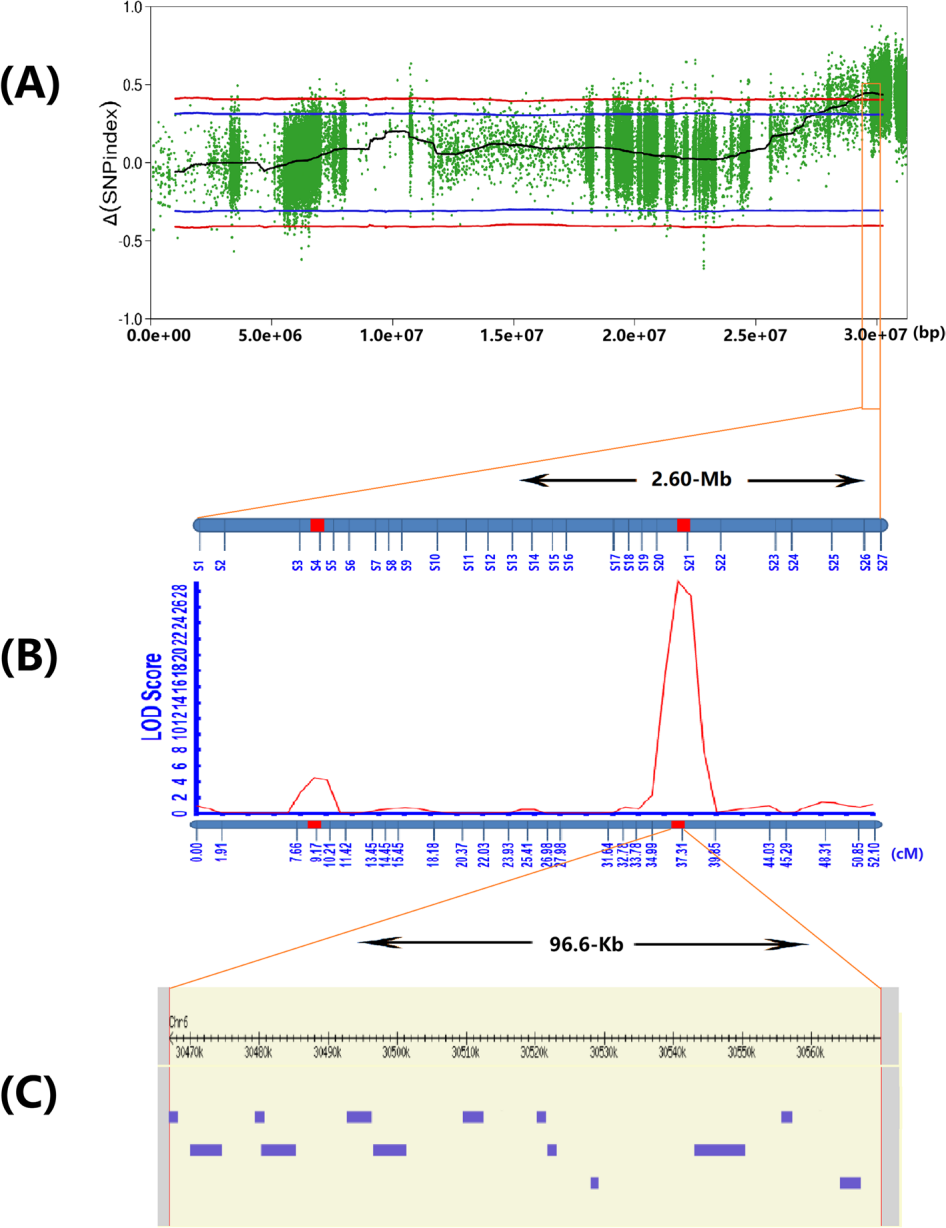
Further mapping of the *qCTS6*. **A** The Manhattan diagram shows the location of the *qCTS6* on chromosome 6. The number on the horizontal coordinate represents the physical position (bp) on the chromosome. **B** *qCTS6* detected by using the inclusive composite interval mapping (ICIM) module of QTL IciMapping 4.2. Linkage map based on SNP markers. The vertical lines on the blue bar represent the name of the SNP marker (all SNP marker information is given in Table S9). The LOD scores are shown with two distinct peaks corresponding to CTS. **C** Thirteen genes in the *qCTS6* region were obtained from annotation information on the Nipponbare genome

Because the 2.60-Mb region contained a large number of genes, we performed fine mapping of *qCTS6* based on the base-variation information of the 2.60-Mb region to identify effective SNPs that might be associated with CTS according to the following criteria: Δ(SNP-Index) values >0.48, which would be significantly higher than the statistical upper bound of the CIs under the null hypothesis of “no QTLs at the *P* < 0.01 level”; and the selected SNP represents a non-synonymous mutation uniformly distributed within that region. We ultimately captured 27 excellent nSNPs within the *qCTS6* interval, with these used for genotype scanning of the 460 lines and to obtain a *qCTS6* linkage map (Fig. 3B). We used a logarithm of the odds (LOD) value >3.0 as a threshold for consecutive occurrence to determine the existence of the QTL. We then analyzed the seedling SR of the 460 lines under cold stress by linkage mapping. *qCTS6* was simultaneously linked with SR and anchored to the 96.6-kb interval between 30,467,391 bp and 30,563,973 bp (Fig. 3B), thereby explaining 25.83% of the phenotypic variation in the SR (Table 2). The positive allele of *qCTS6* was contributed by DF104, and CT6 was optimized to a physical interval of 96.6 kb. Other QTL intervals (29,739,644–29,771,312 bp) with minor effects (explaining 3.17% of the phenotypic variation) were not used in subsequent analysis due to their lower LOD value.

**Table 2:** Identification of *qCTS6* for cold tolerance at seedling stage by linkage analysis

Trait	Chr	Position (cM)	LOD	PVE (%)	Add	LeftCI	RightCI
SR	6	9	4.46	3.18	1.45	8.5	10.5
SR	6	37	29.23	25.83	-4.12	36.5	37.5

*Chr* chromosome, *PVE* phenotypic variation explained

### Putative candidate genes for *qCTS6*

The 96.6-kb interval was intercepted from annotation information of the Nipponbare genome (http://rice.plantbiology.msu.edu/), resulting in capture of 13 annotation genes (Fig. 3C). Except for *FON1* [32] or the functional genes in the number of cloned flower organs, no other genes were evaluated. Previous studies reported important genes or gene families, such as those encoding pseudouridine synthase family protein [33], C3HC4 [34], hypothetical protein [18], protein phosphatase 2C [35], and the bZIP family transcription factor (TF) [36–38], related to CT in rice or other crops. Although similar genes or proteins were included in the list of 13 candidate genes, the 10 genes obtained from Gene Ontology (GO) analysis were not annotated with the phrase “response to cold”. The most enriched terms related to biological process, molecular function, and cellular component ontologies were metabolic processes (i.e., GO:0044267, GO:0008152, and GO:0034641), catalytic activity (i.e., GO:0003824 and GO:0140096), and cell (i.e., GO:0005623 and GO:0044464), respectively (Table S5 and Figure S1). Further, the 96.6-kb region on chromosome 6 harbors 24 SNPs located in the coding region between the parental lines and a Δ(SNP-Index), G-value, ED, and two-tailed Fisher’s exact test *P*-value higher than the statistical confidence at *P* < 0.01. Additionally, 89 SNPs and 18 insertions were located in the intergenic, intronic, untranslated, upstream, and downstream regions. Seven of 13 genes carried 13 nSNPs (Table S6), with four nSNPs identified using both parents as a reference and affecting four candidate genes encoding pseudouridine synthase (Os06g0717400), protein phosphatase 2C (Os06g0717800), a hypothetical protein (Os06g0718000), and a bZIP TF (Os06g0719500) (Table 3). As noted, these genes might play an important role in responding to cold stress in plants. Based on this finding, we identified the four final possible candidate genes using the following three approaches: the possible relationship between gene response and cold stress, non-synonymous mutations in the protein-coding region, and participation of the gene products in specific metabolic pathways that affect CT.

**Table 3:** Identification of SNPs in putative candidate genes for CTS

Gene ID	Position	Amino_acid^**a**^	DF-104 base	DN-430 base	T-pool base	S-pool base	Δ (SNP-index)	G-Value	ED	Fisher exact test	Structure_type	Function
Os06g0717400	30484096	L/S(-)	G	A	G	A	0.71	55.54	1.02	4.97E-13	exonic	hypothetical conserved gene
Os06g0717800	30499155	T/T(-)	A	G	A	G	0.47	21.54	0.44	9.03E-06	exonic exonic	protein phosphatase 2C
	30499218	I/M(-)	C	T	C	T	0.58	33.47	0.68	1.73E-08		
Os06g0718000	30512105	Q/R(-)	C	T	C	T	0.29	8.52	0.17	0.004493	exonic	Hypothetical protein
Os06g0719500	30563973	F/Y(+)	A	T	A	T	0.60	18.83	0.72	4.71E-05	exonic	bZIP transcription factor

^a^ "/" indicates the amino acid before the mutation, the letter after the "/" indicates the amino acid after the mutation, "-" indicates the transcript located on the antisense strand, and "+" indicates the transcript located on the reference genome

**Fig. 4:**
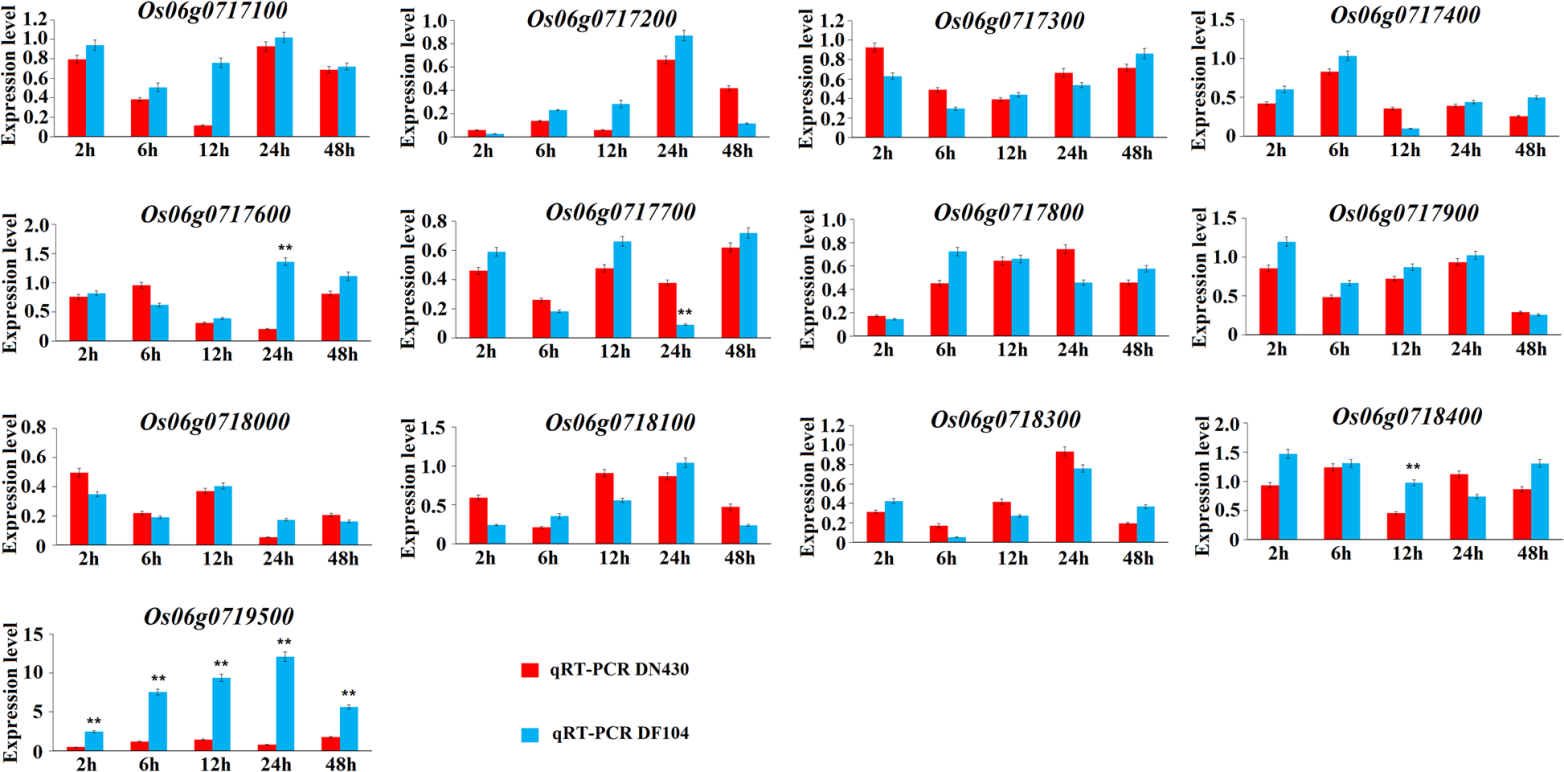
Expression levels of the 13 candidate genes in DN430 and DF104 in response to cold stress and measured by qRT-PCR. **P < 0.01, Student’s t test

**Fig. 5:**
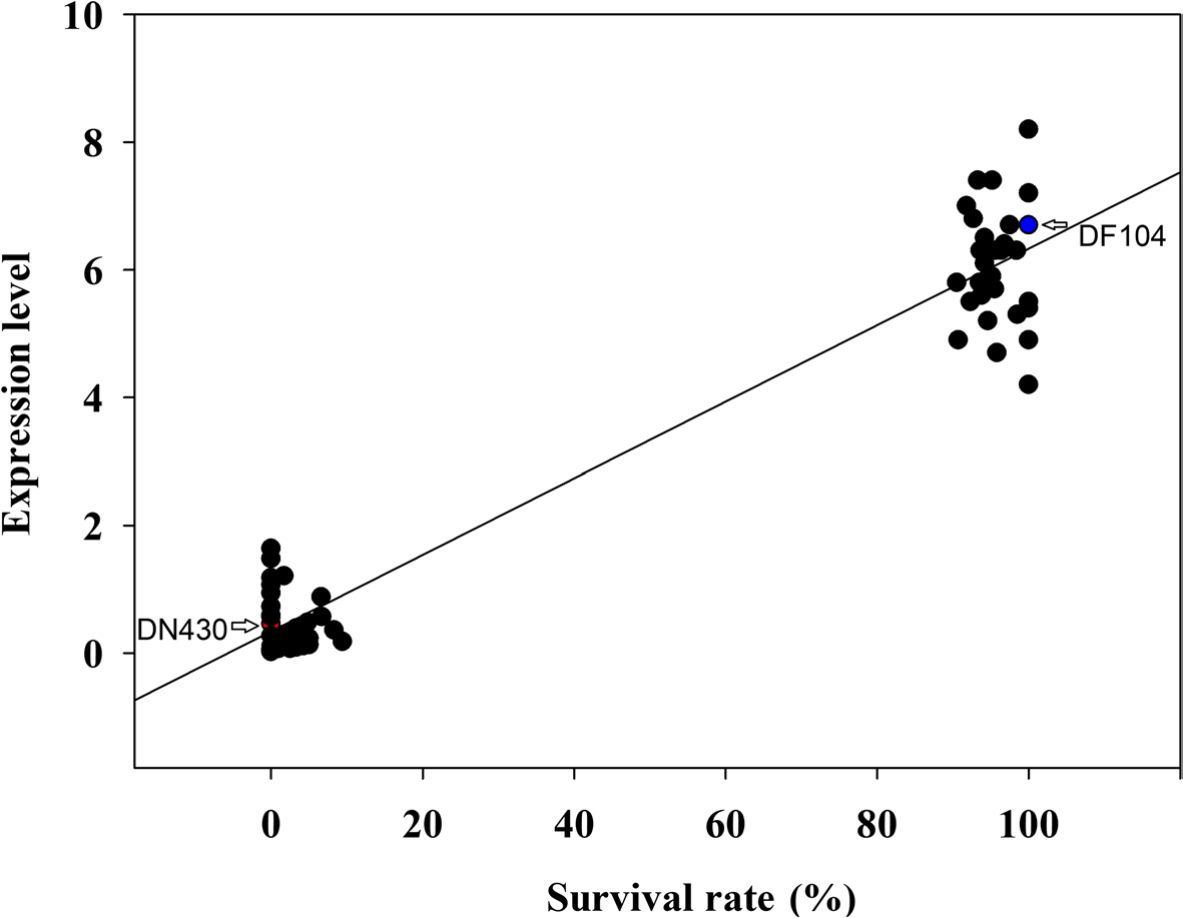
Correlation between OsbZIP54 expression and SR in 30 CT lines and 30 cold-sensitive lines following cold treatment

To identify strong candidate genes in *qCTS6*, we analyzed the expression levels of 13 genes under cold stress and normal conditions, revealing four genes showing significant changes in expression under cold stress. Among these, Os06g0761700, Os06g0717700, and Os06g0718400 levels differed significantly at a single time point, with only Os06g0719500 showing a strong induction of cold-stress response in parents with extremely strong CT (Fig. 4) and suggesting Os06g0719500 as a possible candidate gene for *qCTS6*. By contrast, under normal conditions, all 13 candidate genes showed no significant changes in expression (Figure S2). Notably, Os06g0719500 encodes the bZIP family TF annotated as *OsbZIP54*, with the SNP marker *SNP-630563973* located in this coding region. Moreover, a strong correlation between mRNA levels and CT in these 60 lines indicated the regulatory function of *OsbZIP54* on CT (Fig. 5). These findings suggested *OsbZIP54* as a possible candidate CT gene on *qCTS6* for further analysis.

### Haplotype analysis of candidate genes

**Fig. 6:**
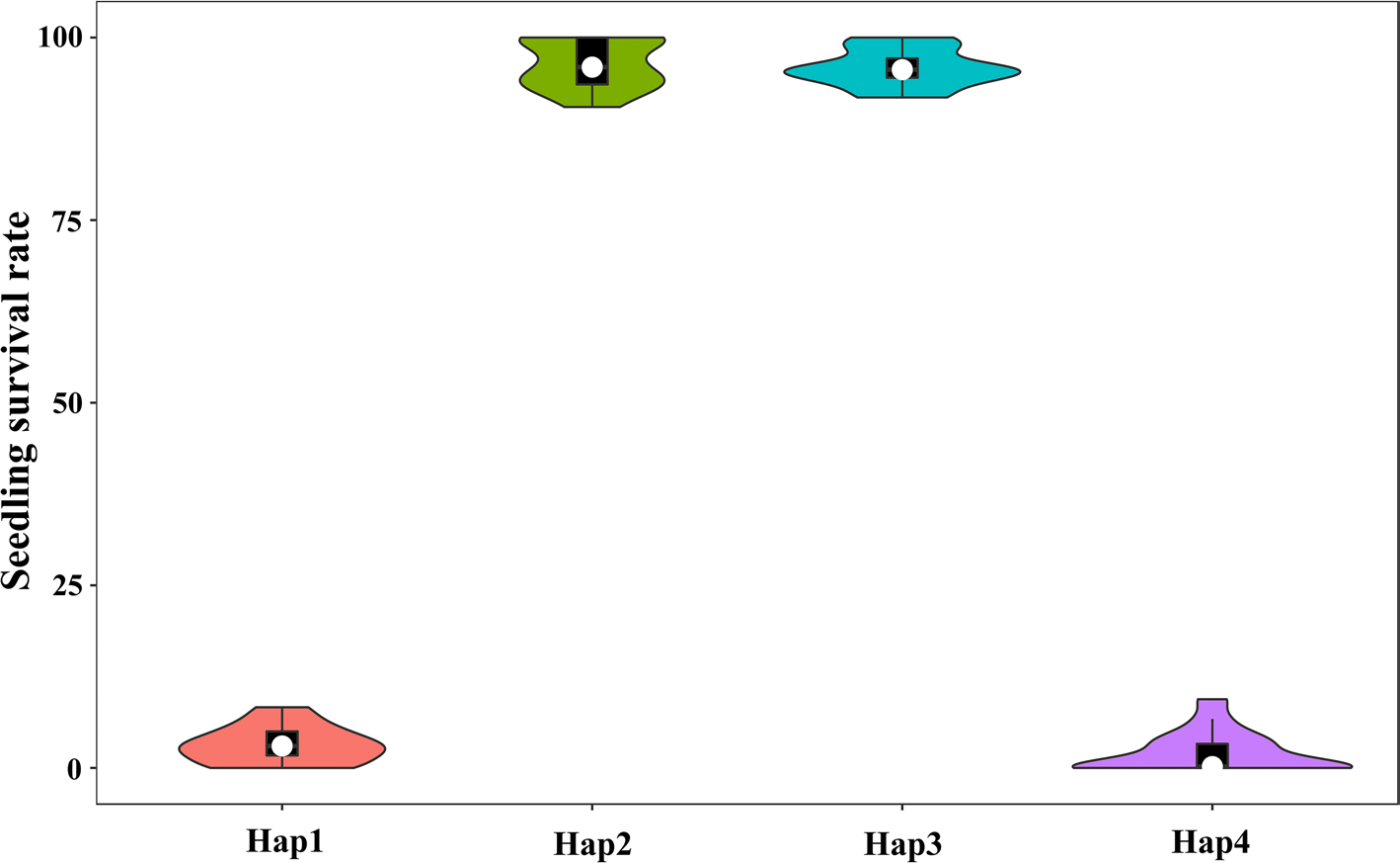
Haplotype–trait violin plots. Hap1 (TG), Hap2 (AA), Hap3 (AG), Hap4 (TA)

To reveal the significance of variations in the *OsbZIP54* coding region, we selected 60 lines from the T-pool and S-pool, respectively, and sequenced their *OsbZIP54* genes by Sanger sequencing. We identified two SNPs (SNP-30563973 and SNP-30565007) on the *OsbZIP54* exon and intron (Figure S3), respectively, with no polymorphism detected in the promoter region between the two parents (Figure S4). To confirm the association of haplotypes with the studied traits, violin plots were generated to assess trait distribution within haplotype groups (Fig. 6) We then found that 30 CT and 30 cold-sensitive lines showed consistent isolation in the +376-bp (T>A) coding region of *OsbZIP54* (Table S7), suggesting that this might lead to phenotypic differences in CT. Moreover, the other SNP (SNP-30565007) was not associated with CT, as *OsbZIP54* was isolated from the japonica rice variety DF-104. To clarify the distribution of +376-bp (T> A) in rice, we used data from the 3010 Rice Genomes Project and found that only 275 japonica rice genomes carried +376-bp (T>A), whereas indica rice genomes harbored >1600 instances. The proportion of *OsbZIP54* functional variation in indica rice and japonica rice was 5.8:1, indicating that +376-bp (T>A) was a rare variation in japonica rice as compared with indica rice. We subsequently analyzed the variation types of *OsbZIP54* in 295 northern japonica rice (Table S8), finding no instance of +376-bp (T>A) in the samples. This suggested that +376-bp (T>A) was a natural variation; therefore, +376-bp (T>A) can be used as a special rare functional variation in DF104. However, it cannot be ruled out that other japonica *OsbZIP54* might harbor a similar single-base variation.

## Discussion

Rice is a staple food for ~50% of the world population [39]. Due to its origin in tropical and subtropical regions, rice is more sensitive to cold stress than other cereal crops. Notably, rice seedlings are sensitive to low temperatures in early Spring in temperate and subtropical zones, as well as in high-elevation areas, leading to delayed growth of seedlings, yellowing, withering, reduced tillers, and hindered growth [2]. To locate the major QTLs of CT, we used several approaches to obtain the most reliable QTL interval. First, we used a large population to identify their phenotypes under low-temperature conditions, thereby obtaining accurate CT phenotypes for each line. Second, two DNA pools with strict CT phenotypic differences were used to perform QTL-seq analysis. Moreover, we used four bioinformatics analysis approaches to map QTL regions at the 99% significance level. Finally, two highly significant peaks (*qCTS3* and *qCTS6*) were detected on chromosomes 3 and 6, with the former mapped between 1.50 Mb and 4.08 Mb and the latter between 28.44 Mb and 31.04 Mb. QTLs and genes for CT on chromosomes 3 and 6 have been previously reported. The QTL *qLVG3* was studied for CT at the bud stage of rice according to the ‘Nipponbare’ reference genome (chromosome 3 at a position 5.92-Mb away from *qCTS3*) [40]. *qCTS3* was located in the 1,500,000- to 4,080,000-bp region, and *CDPK13* [41] and *OsCDPK13* [42] were detected in this region. In chromosome 6, the location of *qCTS6* in the present study completely overlaps with the previously reported region of *qLVG6* and is linked with CT during the budburst period [40]. Additionally, *qRCT6b*, a QTL controlling CT at the reproductive stage, was ~23.00-Mb distant from *qCTS6*. Moreover, we found no CT QTLs related to the seedling stage on chromosome 6 and no cold-response gene has been reported within the *qCTS6* interval. Notably, implementation of three algorithms [∆(SNP-Index), ED, and G-value] resulted in a higher peak value for *qCTS6* (Table S3) than that for *qCTS3*. Furthermore, compared with *qCTS6*, *qCTS3* was not associated with Fisher’s exact test. This suggested the presence of a significant difference in the allele ratio between the two mixed pools. Therefore, we considered *qCTS6* as the most significant target for CTS and exploration of candidate genes.

Using the strategy of traditional QTL mapping combined with QTL-seq, we mapped the *Cf-10* gene to a 790-kb region in *Cladosporium fulvum* [43], and *GaFzl* was mapped to a 70-kb region containing seven annotated genes [44]. Therefore, we believe that strategies combining QTL-seq and traditional linkage mapping for uncovering very narrow candidate regions are well suited for faster targeting of a target gene. To screen potential candidate genes in the *qCTS6* interval, we combined QTL-seq and fine mapping, which enabled reduction of the number of candidate genes within the *qCTS6* interval defined by QTL-seq from 393 to 13 genes. Among these 13 genes, seven have 13 functional base variations. Furthermore, the expression level of *OsbZIP54* in the two parents showed that it was strongly induced after cold treatment. Expression analysis of *OsbZIP54* in 60 lines with differences in extreme CT revealed a correlation between CT and expression level (Fig. 5). Additionally, sequence analysis revealed two SNP differences in the coding-sequence region of *OsbZIP54*, in which +376-bp (T>A) is a functional variant, and that play a major role in determining the difference in CT (Table S7). However, as a newly identified CT regulator, the signaling-transduction system and the downstream pathways of *OsbZIP54* remain unclear. DF104 carries a rare exonic SNP, and *OsbZIP54* was highly induced under cold conditions, suggesting that the SNP might be involved in transcriptional regulation of *OsbZIP54*, which warrants further exploration. Interestingly, despite the strong expression of *OsbZIP54* induced by cold stress, the promoters of DF-104 and DN-430 do not harbor polymorphisms. A previous study reported that *bZIP73* is induced and upregulated by cold stress, and that there is a SNP difference at the 511-bp position of the coding region that determines CT of the rice [36]. In the present study, we identified no polymorphisms the *bZIP54* promoter. However, another study showed that bZIP73–bZIP71 interactions might facilitate transcriptional activation of *bZIP73*, which subsequently confers a cold-signal switch in rice. This provides evidence that a possible reason for upregulated *OsbZIP54* expression is cold stress. However, the expression level of *CTB4a* under cold stress is affected by promoter mutations and functional SNPs [45]; therefore, the genetic mechanism of rice in response to cold stress is complex, and significant alterations in the expression of genes under cold stress are not solely determined by their respective promoters. Next, we will further clone the alleles of the candidate gene, construct expression vectors for plants and yeast, and express these vectors in rice protoplasts, yeast, and *Arabidopsis* to compare the effects of this natural allele variation on gene expression and subcellular localization under cold stress. The biological functions of *OsbZIP54* and its associated genomic variations need to be confirmed using gene editing and high-efficiency overexpression transformation systems. Although we found correlations between the nSNP in Os09g0444200 and the studied phenotypic traits, this is insufficient to assign a CT phenotype to a single SNP. By contrast, we also believe that a favorable allelic variant in one key gene is insufficient to provide CT, and that the final CT phenotype of the plant should be considered a result of a combination of favorable allelic variations from different key genes.

CT is a complex trait controlled by multiple genes [46], and those genes isolated thus far can be divided into two different types. One includes genes with specific functions and that participate in systemic metabolism to defend against cold stress [47]. For example, *OsLTPL159* enhances the CT of rice at the early seedling stage by decreasing the toxic effect of reactive oxygen species, enhancing cellulose deposition in the cell wall, and promoting osmolyte accumulation [21]. Additionally, *CTB4a*, as a leucine-rich repeat receptor-like kinase that positively regulates ATP activity and content under cold stress by interacting with the β subunit of ATP synthase, thereby increasing pollen fertility [48]. The second type comprises genes that regulate gene expression during stress responses (i.e., signaling components and TFs) [47]. Together with their target genes, TFs can serve as regulators of signal transduction to activate or inhibit genes involved in cold-stress response. Therefore, TFs are excellent candidates for modifying complex traits of crops [49]. Conversely, there are 91 bZIP family member genes in rice [50], several of which are involved in the regulation of CT. Notably, as a transcriptional activator, the expression of *OsbZIP52* was strongly induced at low temperature (4°C) [51]. Other bZIPs, such as *bZIP73*, interact with *bZIP71* to regulate the level of abscisic acid and the balance of active oxygen, thereby improving the tolerance of rice to low temperatures [36]. However, in northern japonica rice, the potential of bZIP TFs in CT remains to be explored. In the present study, we identified a rare variation in *OsbZIP54* carried in northern China japonica rice, and haplotype events did not occur in 295 northern cultivars (Table S8). Additionally, the 3010 Rice Genome Project database shows that *OsbZIP54* has a very small occupancy rate in japonica rice as compared with indica rice. A rare thymine variation on the *OsbZIP54* exon was found in DF104, which could serve as a genomic marker for improved CT. Moreover, the molecular mechanism of the interaction between *OsbZIP54* and its targeted promoter elements, downstream genes, and cofactors warrant further investigation.

## Conclusion

In conclusion, study of the genetic basis of CTS is important for breeding purposes. In this study, we used QTL-seq and linkage-map analysis to identify the *qCTS6* QTL related to rice CT. Experimental verification by qRT-PCR and haplotype analysis indicated *OsbZIP54* in the *qCTS6* region as a candidate gene. Furthermore, the results showed that the Dongfu104 allele exhibiting a specific cold-induction expression pattern could serve as an important molecular marker for rice improvement.

## Materials and methods

### Plant materials

We used two japonica varieties [cold-sensitive female parent Dongnong 430 (DN430) and cold-tolerant male parent Dongfu104 (DF104)] as parental lines to develop the 460 F_2:3_ population. These materials were obtained from Northeast Agriculture University (Harbin, China).

#### CTS evaluation

The seeds were incubated at 40°C for ~36 h to break dormancy and then soaked in deionized water at 30°C for ~72 h for germination. The germinated seeds were transplanted into 96-well PCR plates for seedling growth in a greenhouse environment (26°C day/22°C night temperature, 12-h/12-h light/dark cycle, and 80% relative humidity). When the seedlings grew to trifoliate, they were treated to a low temperature of 9°C/10 days in an incubator, and all 460 F_2:3_ seedlings were allowed to resume growth at 28°C for 10 days to investigate the SR (measured as the percentage of total seedlings that survived relative to the total number tested) of 60 plants per replicate. Three biological replicates were performed.

### Construction of segregating pools

All young leaves of 460 F_2_ individuals were collected separately for total genomic DNA extraction using a cetyltrimethylammonium bromide method [52], with minor modifications. The genomic DNA of 30 extremely CT (ECT) and 30 extremely cold-sensitive (ECS) individuals were selected as two bulked pools according to the SR of the F_2:3_ population (range: 0–100%). [To simplify the following description, we abbreviate DF104 as T, DN430 as S]. Isolated DNA was quantified using a Nanodrop 2000 spectrophotometer (Thermo Fisher Scientific, Waltham, MA, USA). All DNA from the 30 ECT and 30 ECS plants were quantified on a Qubit 2.0 fluorimeter (Life Technologies, Carlsbad, CA, USA), and equal amounts of DNA from the ECT and ECS plants were mixed.

### NGS sequencing and BSA-seq analysis

Total genomic DNA was extracted from bulked pools, and at least 3 μg genomic DNA was used to construct paired-end libraries with an insert size of 500 bp using the paired-end DNA sample prep kit (Illumina, San Diego, CA, USA). These libraries were sequenced using the HiSeq X10 (Illumina) NGS platform at Genedenovo (Guangzhou, China). Quality trimming is an essential step in generating high-confidence variant calling. Raw reads were processed to obtain high-quality clean reads according to three stringent filtering standards: 1) removing reads with ≥10% unidentified nucleotides; 2) removing reads with > 50% bases having phred quality scores ≤ 20; and 3) reads aligned to the barcode adapter.

To identify SNPs and indels, filtered reads were aligned to the Nipponbare reference genome sequence [53] using the Burrows–Wheeler Aligner (v.0.7.16a-r1181) with parameter ‘mem -M’; -M is an option used to mark shorter split-alignment hits as secondary alignments [54]. Variant calling was performed using the GATK UnifiedGenotyper (v.3.5; https://gatk.broadinstitute.org/hc/en-us/community/posts/360073637011-UnifiedGenotyper-in-GATK4). SNPs and indels were filtered using the GATK VariantFiltration function with proper standards (-Window 4, -filter "QD < 4.0 || FS > 60.0 || MQ < 40.0 ", -G_filter "GQ < 20"). All mutations were annotated for genes and functions, as well as genomic regions, using ANNOVAR [55]. Association analysis was performed using the SNP-Index [23], ∆(SNP-Index) [56], calculation of the G statistic [57, 58], ED [59], and two-tailed Fisher’s exact test [60] based on the SNPs. Average values were calculated using a 2000-kb sliding window with a step size of 20 kb and discarding of windows with less than 10 SNP/Indel. If the number of SNPs was insufficient, the results of this window were merged into the next window. The overlapping interval of the four methods was considered as the final QTL interval of CT.

### Development of SNP markers and narrowing the candidate interval

To develop markers to validate the QTL-seq results and narrow the candidate region, 27 nSNPs with significant peaks were identified based on the CDS sequence of the two parents in the *qCST6* region under the four association-analysis algorithms [∆(SNP-Index), G-value, ED, and Fisher’s exact test] and were considered as markers for fine mapping. Primer Premier 5.0 (Premier Biosoft International, Palo Alto, CA, USA) was used to design primers (Table S9). All markers were selected for polymorphisms between parents, and genotyping of the 460 individuals was performed using the polymorphic markers, which were used to construct the linkage map and narrow the candidate region using the ICIM module of QTL IciMapping (v.4.2; http://www.isbreeding.net). The threshold of the LOD score for declaring the presence of a significant QTL was determined by the permutation test with 1000 repetitions at *P* < 0.01.

### Prediction of candidate genes for the CTS

To predict possible candidate genes for the CTS, the following three strategies were comprehensively considered. First, candidate gene prediction was performed to compare the DNA sequences of genes within the QTL regions using the whole-genome DNA re-sequencing database of the parents. In the QTL regions, we focused on open reading frames with non-synonymous mutant SNPs between the two parents. Second, candidates with the highest probability of success were reselected according to their functional annotations and used to screen the rice genome database (http://rice.plantbiology.msu.edu/). Third, the GO [61] and Kyoto Encyclopedia of Genes and Genomes [62] databases were used as references for the evaluation of candidate genes using BLAST software [63].

### Verification of the expression of candidate genes

Seedling leaves of DN430 and DF104 were collected at 2 h, 6 h, 12 h, 24 h, and 48 h after cold treatment in three replicates, frozen in liquid nitrogen, and stored at −80°C until total RNA extraction. Control plants were also collected and stored similarly. qRT-PCR was used to quantify the expression levels of candidate genes under cold treatment. Total RNA was extracted from rice tissues using TRIzol reagent (Thermo Fisher Scientific) and treated with DNase I to eliminate any DNA contamination. RNA quality was assessed by electrophoresis and stored at −80°C until use. First-strand cDNA (10 μL) was synthesized according to the instructions for the PrimeScript RT Master Mix (Takara Biomedical Technology (Beijing) Co., Ltd., Beijing, China). Primers were designed with Primer Premier v. 5.0 (Premier Biosoft International) and are listed in Table S9. The housekeeping gene *actin1* (Os05g36290) was used as an internal control [64], and qRT-PCR was performed using a LightCycler 2.10 system (Roche, Basel, Switzerland) with 2× SYBR Green I PCR Master Mix (Thermo Fisher Scientific). qRT-PCR analysis was performed as previously described [65].

### Diversity analysis of candidate genes

Haplotypes of candidate genes were analyzed, as follows: 30 T-pool, 30 S-pool, and 295 China northern japonica rice. The 295 rice varieties were from the Heilongjiang, Jilin, and Liaoning provinces on China and other countries, including Japan, the Republic of Korea, the Democratic People’s Republic of Korea, and Russia [66]. Primer sequences were designed for candidate genes at SNP sites between parents, and the DNA fragments of the sequence in 60 lines were amplified by PCR to determine haplotype statistics of the distribution of target genes. All specific primer sequences are provided in Table S2. The PCR reaction mixture had a total volume of 20 μL and comprised 1.5 μL of forward primer (10 μm), 1.5 μL of reverse primer (10 μm), 2 μL of genomic DNA (50 ng/μL), 5 μL of ddH_2_O, and 10 μL of Pfu master mix (Cwbio, Beijing, China), which included Taq DNA polymerase, PCR buffer, Mg^2+^, and dNTPs. The PCR reaction was performed in an Eppendorf 5333 Mastercycler (Eppendorf, Hamburg, Germany) using the same protocol as that used for qRT-PCR [65]. The products were examined by 1% agarose gel electrophoresis. Direct sequencing of PCR products was performed by BGI Life Technology Co., Ltd. (Shenzhen, China). Haplotype trait data were plotted in the ggplot2 R package (ggplot2: elegant graphics for data analysis; Springer, NY, USA).

### Statistical analysis

The difference between the two parents and progeny for CT was detected using SPSS18.0 software. Data represent means ± standard deviations. Correction analysis was performed to evaluate correlations between gene expression and seedling survival rates using SigmaPlot software (v.12.5; Systat Software, San Jose, CA, USA).

## Supplementary Information

**Additional file 1: Figure S1**. Clustering map of GO annotation of genes in 96.6- Kb regions. The abscissa is the content of GO categories, and the left of the ordinate is the number of genes. This figure shows the gene classification of GO secondary functions in the context of all genes in the associated region. **Figure S2**. Expression levels of the 13 candidate genes in DN430 and DF104 under normal condition measured by qRT-PCR. The results were statistically analyzed using Student’s t-test (**, *P*<0.01). **Figure S3**. The CDS region Sequence difference analysis of OsbZIP54. The gene structure of Os07g0569700 and sequence differences in OsbZIP54 between DF-104 and DN-430. Ref is the reference sequence of Nipponbare genome. **Figure S4**. The promoter Sequence difference analysis of *OsbZIP54*. The promoter structure of *OsbZIP54* and sequence differences in *OsbZIP54* between DF-104 and DN-430. Ref is the reference sequence of Nipponbare genome. **Supplemental Table 1**. Number of single nucleotide polymorphisms (SNPs) and InDels detected in samples. **Supplemental Table 2**. Survival rate of parents and F2:3 population under cold treatment. **Supplemental Table 3**. Significant peak statistics driven by four algorithms. **Supplemental Table 4**. Statistics of variation in the 2.60 Mb interval of qCTS6 based on resequencing data. **Supplemental Table 5**. GO annotation result of 96.6-Kb interval. **Supplemental Table 6**. Statistics of variation in the 96.6Kb interval based on resequencing data. **Supplemental Table 7**. The Haplotype analysis of Os06g0719500 in the T-Pool and S-Pool. **Supplemental Table 8**. Base Variation Statistics of 295 North China Japonica Rice in qCTS6 Interval. **Supplemental Table 9**. Primers used in this study.

## Data Availability

The QTL-seq data for this study can be found in the National Center for Biotechnology Information Sequence Read Archive under the accession numbers SSR13319856, SSR13319857, SSR13319858, and SSR13319859 (https://www.ncbi.nlm.nih.gov/bioproject/PRJNA688381).

## Abbreviations

BSA: Bulked segregant analysis
CDS: Coding sequence
CI: Confidence interval
CT: Cold tolerance
CTS: Cold tolerance at the seedling stage
ED: Euclidean distance
ICIM: Inclusive composite interval mapping
LOD: Logarithm of odds
nSNP: Non-synonymous SNP
qRT-PCR: Quantitative reverse transcription PCR
QTL: Quantitative trait loci
SNP: Single-nucleotide polymorphism
SR: Survival rate
TF: Transcription factor
