# Supplementary figures and images for: Combining QTL-seq and linkage mapping to fine map a candidate gene in qCTS6 for cold tolerance at the seedling stage in rice
Source: BMC Plant Biol. 2021 Jun 19;21:278. doi: 10.1186/s12870-021-03076-5 (PMC8214256; doi:10.1186/s12870-021-03076-5)

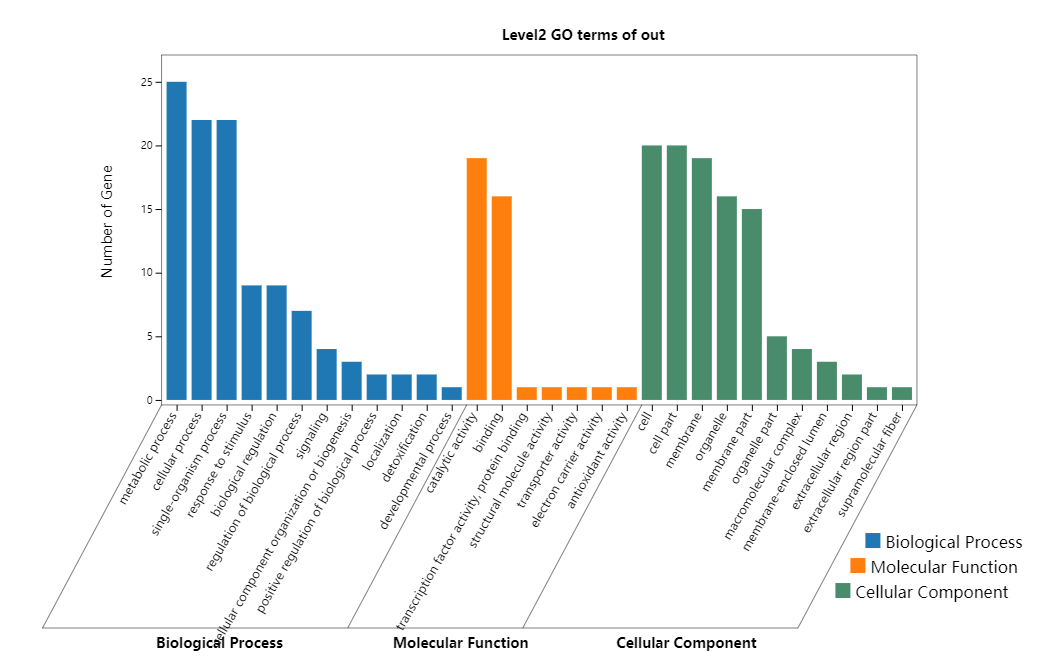

Supplement: Supplementary file 1 — Additional file 1: Figure S1. Clustering map of GO annotation of genes in 96.6- Kb regions. The abscissa is the content of GO categories, and the left of the ordinate is the number of genes. This figure shows the gene classification of GO secondary functions in the context of all genes in the associated region. Figure S2. Expression levels of the 13 candidate genes in DN430 and DF104 under normal condition measured by qRT-PCR. The results were statistically analyzed using Student’s t-test (**, P<0.01). Figure S3. The CDS region Sequence difference analysis of OsbZIP54. The gene structure of Os07g0569700 and sequence differences in OsbZIP54 between DF-104 and DN-430. Ref is the reference sequence of Nipponbare genome. Figure S4. The promoter Sequence difference analysis of OsbZIP54. The promoter structure of OsbZIP54 and sequence differences in OsbZIP54 between DF-104 and DN-430. Ref is the reference sequence of Nipponbare genome. Supplemental Table 1. Number of single nucleotide polymorphisms (SNPs) and InDels detected in samples. Supplemental Table 2. Survival rate of parents and F2:3 population under cold treatment. Supplemental Table 3. Significant peak statistics driven by four algorithms. Supplemental Table 4. Statistics of variation in the 2.60 Mb interval of qCTS6 based on resequencing data. Supplemental Table 5. GO annotation result of 96.6-Kb interval. Supplemental Table 6. Statistics of variation in the 96.6Kb interval based on resequencing data. Supplemental Table 7. The Haplotype analysis of Os06g0719500 in the T-Pool and S-Pool. Supplemental Table 8. Base Variation Statistics of 295 North China Japonica Rice in qCTS6 Interval. Supplemental Table 9. Primers used in this study. [file 12870_2021_3076_MOESM1_ESM.zip › Figure S1.png]

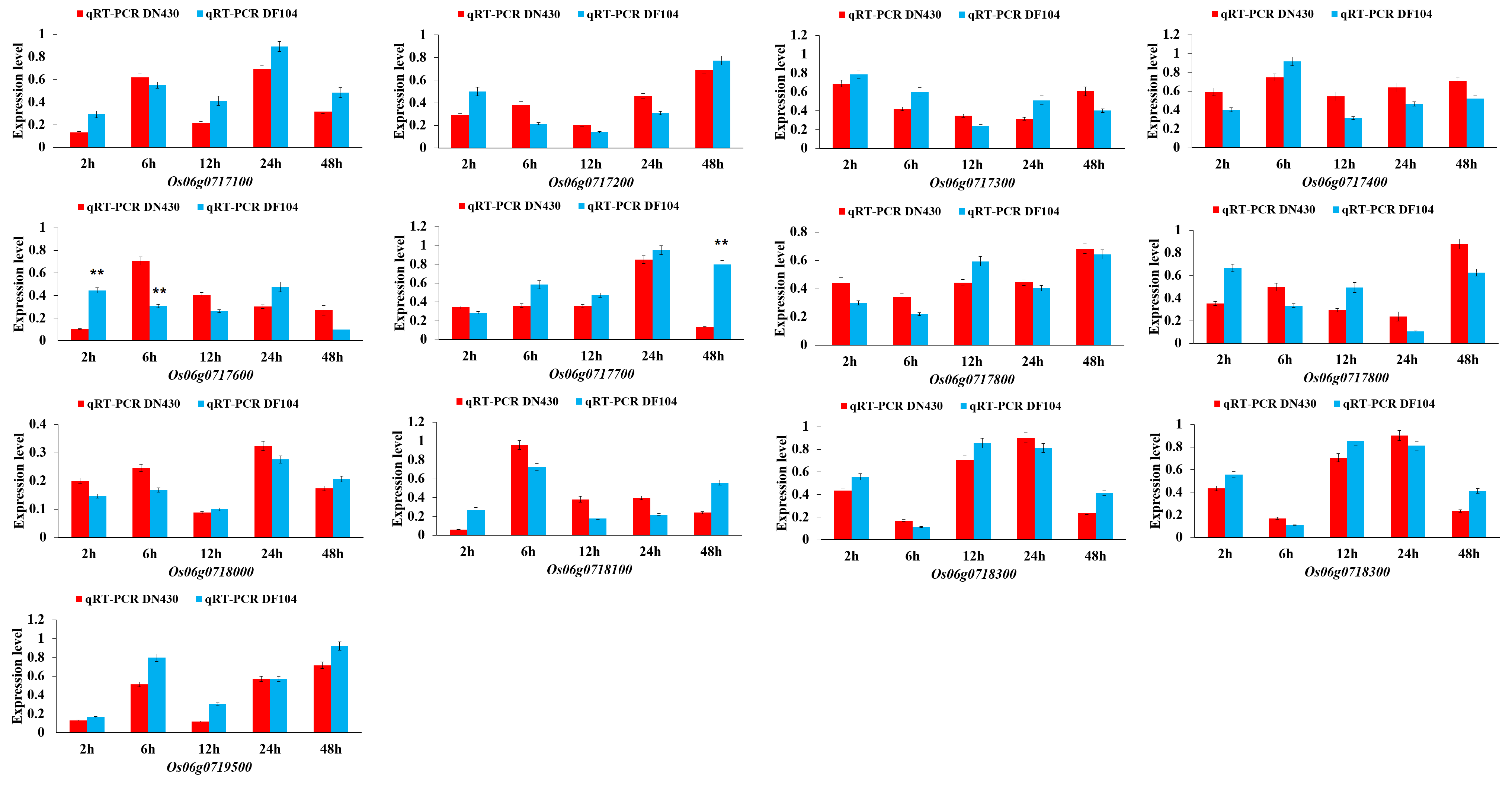

Supplement: Supplementary file 1 — Additional file 1: Figure S1. Clustering map of GO annotation of genes in 96.6- Kb regions. The abscissa is the content of GO categories, and the left of the ordinate is the number of genes. This figure shows the gene classification of GO secondary functions in the context of all genes in the associated region. Figure S2. Expression levels of the 13 candidate genes in DN430 and DF104 under normal condition measured by qRT-PCR. The results were statistically analyzed using Student’s t-test (**, P<0.01). Figure S3. The CDS region Sequence difference analysis of OsbZIP54. The gene structure of Os07g0569700 and sequence differences in OsbZIP54 between DF-104 and DN-430. Ref is the reference sequence of Nipponbare genome. Figure S4. The promoter Sequence difference analysis of OsbZIP54. The promoter structure of OsbZIP54 and sequence differences in OsbZIP54 between DF-104 and DN-430. Ref is the reference sequence of Nipponbare genome. Supplemental Table 1. Number of single nucleotide polymorphisms (SNPs) and InDels detected in samples. Supplemental Table 2. Survival rate of parents and F2:3 population under cold treatment. Supplemental Table 3. Significant peak statistics driven by four algorithms. Supplemental Table 4. Statistics of variation in the 2.60 Mb interval of qCTS6 based on resequencing data. Supplemental Table 5. GO annotation result of 96.6-Kb interval. Supplemental Table 6. Statistics of variation in the 96.6Kb interval based on resequencing data. Supplemental Table 7. The Haplotype analysis of Os06g0719500 in the T-Pool and S-Pool. Supplemental Table 8. Base Variation Statistics of 295 North China Japonica Rice in qCTS6 Interval. Supplemental Table 9. Primers used in this study. [file 12870_2021_3076_MOESM1_ESM.zip › Figure S2.png]

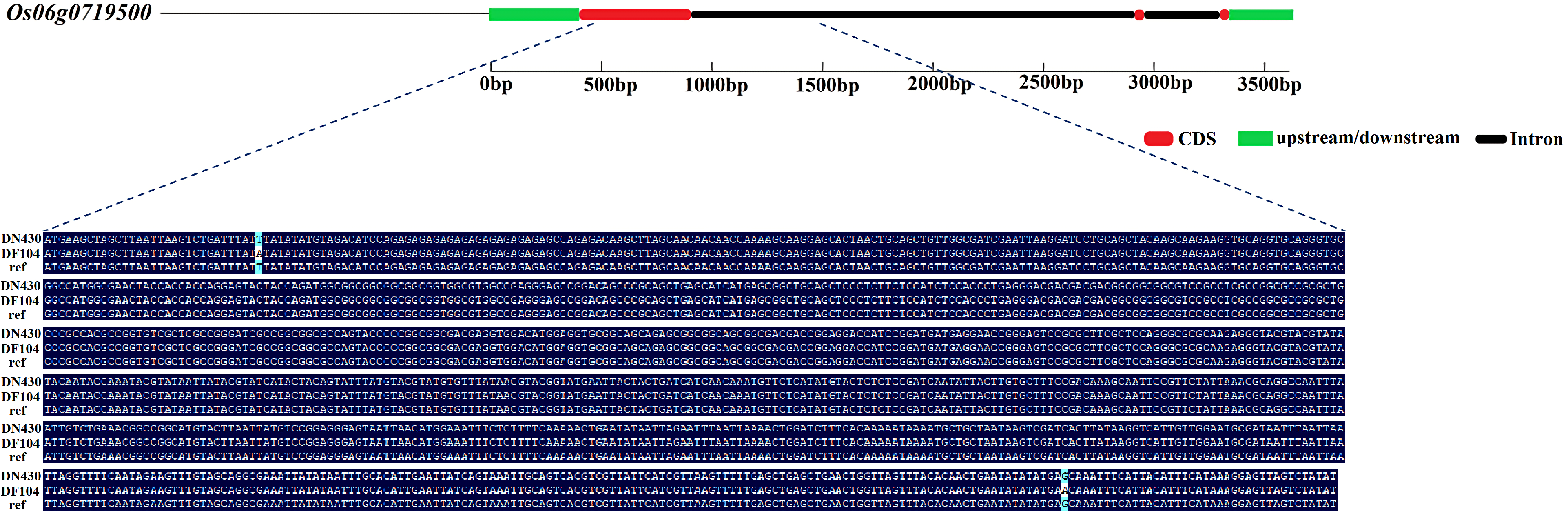

Supplement: Supplementary file 1 — Additional file 1: Figure S1. Clustering map of GO annotation of genes in 96.6- Kb regions. The abscissa is the content of GO categories, and the left of the ordinate is the number of genes. This figure shows the gene classification of GO secondary functions in the context of all genes in the associated region. Figure S2. Expression levels of the 13 candidate genes in DN430 and DF104 under normal condition measured by qRT-PCR. The results were statistically analyzed using Student’s t-test (**, P<0.01). Figure S3. The CDS region Sequence difference analysis of OsbZIP54. The gene structure of Os07g0569700 and sequence differences in OsbZIP54 between DF-104 and DN-430. Ref is the reference sequence of Nipponbare genome. Figure S4. The promoter Sequence difference analysis of OsbZIP54. The promoter structure of OsbZIP54 and sequence differences in OsbZIP54 between DF-104 and DN-430. Ref is the reference sequence of Nipponbare genome. Supplemental Table 1. Number of single nucleotide polymorphisms (SNPs) and InDels detected in samples. Supplemental Table 2. Survival rate of parents and F2:3 population under cold treatment. Supplemental Table 3. Significant peak statistics driven by four algorithms. Supplemental Table 4. Statistics of variation in the 2.60 Mb interval of qCTS6 based on resequencing data. Supplemental Table 5. GO annotation result of 96.6-Kb interval. Supplemental Table 6. Statistics of variation in the 96.6Kb interval based on resequencing data. Supplemental Table 7. The Haplotype analysis of Os06g0719500 in the T-Pool and S-Pool. Supplemental Table 8. Base Variation Statistics of 295 North China Japonica Rice in qCTS6 Interval. Supplemental Table 9. Primers used in this study. [file 12870_2021_3076_MOESM1_ESM.zip › Figure S3.png]

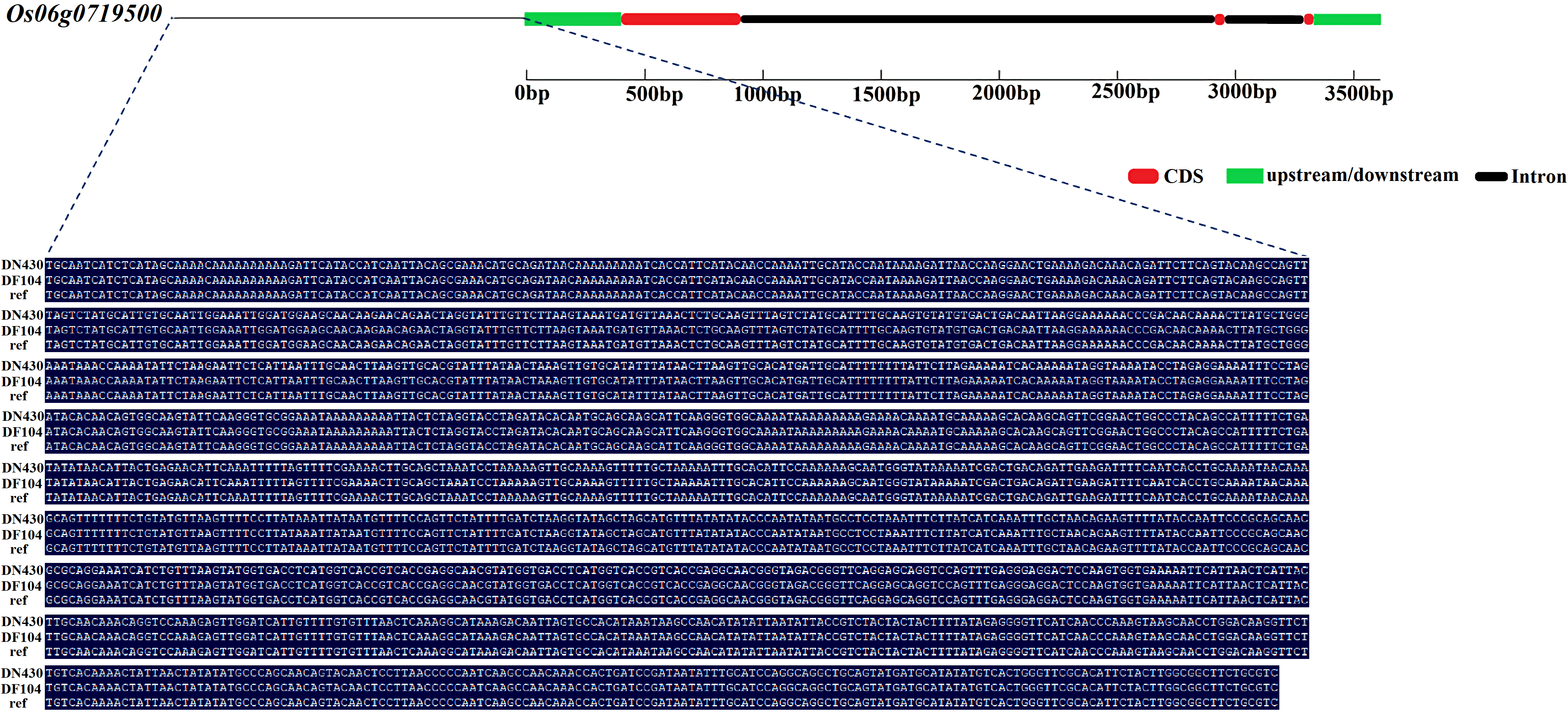

Supplement: Supplementary file 1 — Additional file 1: Figure S1. Clustering map of GO annotation of genes in 96.6- Kb regions. The abscissa is the content of GO categories, and the left of the ordinate is the number of genes. This figure shows the gene classification of GO secondary functions in the context of all genes in the associated region. Figure S2. Expression levels of the 13 candidate genes in DN430 and DF104 under normal condition measured by qRT-PCR. The results were statistically analyzed using Student’s t-test (**, P<0.01). Figure S3. The CDS region Sequence difference analysis of OsbZIP54. The gene structure of Os07g0569700 and sequence differences in OsbZIP54 between DF-104 and DN-430. Ref is the reference sequence of Nipponbare genome. Figure S4. The promoter Sequence difference analysis of OsbZIP54. The promoter structure of OsbZIP54 and sequence differences in OsbZIP54 between DF-104 and DN-430. Ref is the reference sequence of Nipponbare genome. Supplemental Table 1. Number of single nucleotide polymorphisms (SNPs) and InDels detected in samples. Supplemental Table 2. Survival rate of parents and F2:3 population under cold treatment. Supplemental Table 3. Significant peak statistics driven by four algorithms. Supplemental Table 4. Statistics of variation in the 2.60 Mb interval of qCTS6 based on resequencing data. Supplemental Table 5. GO annotation result of 96.6-Kb interval. Supplemental Table 6. Statistics of variation in the 96.6Kb interval based on resequencing data. Supplemental Table 7. The Haplotype analysis of Os06g0719500 in the T-Pool and S-Pool. Supplemental Table 8. Base Variation Statistics of 295 North China Japonica Rice in qCTS6 Interval. Supplemental Table 9. Primers used in this study. [file 12870_2021_3076_MOESM1_ESM.zip › Figure S4.png]
